# The Final Action on the Wilcox Bill

**Published:** 1892-08

**Authors:** 


### THE FINAL ACTION ON THE WILCOX BILL.

We published in our last issue a full report of the action of the two committees of the Maryland State Dental Society and the Odontological Society of Pennsylvania in connection with this bill, now before the Senate, it having passed the House of Representatives. The joint committee had an interview with the members of the Senate committee, of which the Hon. Eugene Hale is chairman. The subject was fully discussed and the justice of the claims of the dentists conceded, but the Superintendent of the Census, Hon. Robert T. Porter, objected to the following amendment to the bill proposed by the committee:

"*Provided that nothing in this act shall be construed as authority to collect statistics from professional men, such as lawyers, physicians, and dentists.*" The objection was founded on the fact that the bill was primarily intended to meet difficulties in which dentists were not involved, and should it go back to the House its final passage would be endangered.

The following decision was finally reached: That if the committee would waive further objection to the bill as at present framed, Superintendent Porter should state in writing, in the presence of the Senate committee, the course he proposed to follow. We append a copy of the agreement.

Washington, D. C., June 27, 1892.

*The Superintendent of Census agrees with the representatives of the dental associations present at a meeting of the Senate Committee on the Census, to carry out the spirit and purpose of the amendment presented by the said associations at the meeting, and which appears in the paper marked "A," herewith attached.*

(Signed) Robert P. Porter,
*Superintendent of Census.*

Dentists throughout the country will henceforth know how to act when called upon by the agents.

The joint committee deserve, and will, we are sure, receive the thanks of the profession for the prompt and successful completion of this difficult work.

